# Do side effects of antidepressants impact efficacy estimates based on the Hamilton Depression Rating Scale? A pooled patient-level analysis

**DOI:** 10.1038/s41398-021-01364-0

**Published:** 2021-04-27

**Authors:** Fredrik Hieronymus, Alexander Lisinski, Elias Eriksson, Søren Dinesen Østergaard

**Affiliations:** 1 Department of Pharmacology, Sahlgrenska Academy, University of Gothenburg, Gothenburg, Sweden; 2 Department of Clinical Medicine, Aarhus University, Aarhus, Denmark; 3 Department of Affective Disorders, Aarhus University Hospital, Aarhus, Denmark

**Keywords:** Depression, Neuroscience

## Abstract

The Hamilton Depression Rating Scale (HDRS-17) measures symptoms that may overlap with common antidepressant side effects (e.g., sexual dysfunction), thus making it possible that side effects of antidepressant treatment are erroneously rated as symptoms of depression, and vice versa. This study uses patient-level data from previously conducted antidepressant treatment trials to assess whether side effect ratings co-vary with HDRS-17 ratings. Data from all HDRS-17-rated, industry-sponsored pre- and post-marketing trials (*n* = 4647) comparing the serotonin and noradrenaline reuptake inhibitor, duloxetine, to placebo and/or to a selective serotonin reuptake inhibitor were pooled; three studies, which utilised sub-therapeutic doses, did not have symptom-level ratings available and could not be included. Severity was assessed for side effects related to sleep, somatic anxiety, gastrointestinal function, and sexual dysfunction. Analysis of covariance was used to assess the relation between these side effects and ratings of relevant HDRS-17-derived outcome parameters. Side effects related to sleep, somatic anxiety and sexual dysfunction significantly and exclusively associated with higher scores on HDRS-17 items measuring the corresponding domains. Side effects related to gastrointestinal function associated with higher HDRS-17 item scores on all assessed domains. Treatment outcome was significantly related to side effect severity when assessed using HDRS-17-sum (beta 0.32 (0.074), *p* < 0.001), but not when the HDRS-6-sum-score (beta 0.035 (0.043), *p* = 0.415) or the depressed mood item (beta 0.007 (0.012), *p* = .527) were used as effect parameters. That some HDRS-17 items co-vary with common antidepressant side effects suggests some of these adverse events are counted twice, potentially leading to an underestimation of antidepressant efficacy.

## Introduction

It is desirable that a rating scale accurately reflects the underlying severity of the condition it aims to depict, as well as how this severity is influenced by treatment^1,2^. This has since long been suggested not to be the case for the effect parameter most widely used in trials of antidepressants, namely the sum of the 17 items of the Hamilton Depression Rating Scale (HDRS-17-sum)^3–6^. One potential drawback of the HDRS-17-sum as a measure of depression severity is that some of the items included with the intention to quantify depressive severity may also capture common antidepressant side effects (SEs)^7,8^.

Selective serotonin reuptake inhibitors (SSRIs) and serotonin and noradrenaline reuptake inhibitors (SNRIs) are the most commonly prescribed drugs for the treatment of major depression^9^. Among the SEs frequently reported in relation to their use are insomnia, sweating, nausea, constipation, diarrhoea, loss of appetite, ejaculation difficulties, anorgasmia, loss of libido, and weight loss^10–12^. As the HDRS-17 includes three items pertaining to insomnia (items 4–6), one for gastrointestinal symptoms (item 12), one for genital symptoms (item 14), and one for weight loss (item 16)^13^, there is potential for the SEs of SSRIs and SNRIs to be erroneously accounted for as symptoms of depression. Similarly, the somatic anxiety item of the HDRS-17 (item 11), which includes complaints such as dry mouth, micturition difficulties, palpitations, etc., may also reflect common antidepressant SEs. If at hand, such a mechanism would lead to an underestimation of the antidepressant efficacy of these compounds^4,7,14^. Conversely, it is possible that the antidepressant efficacy of compounds with sedative or appetite increasing effects may be erroneously overestimated due to reduced ratings of sleep disturbance or weight loss^15^.

We have previously reported that the HDRS-17 items most likely to reflect common SSRI SEs either do not separate active treatment from placebo, or show significantly larger improvements in placebo-treated patients than in SSRI-treated patients^16,17^. In these studies, however, we did not analyse SE reports. In the present study, we follow up on these findings by including data on SEs related to sleep, somatic anxiety, gastrointestinal function, or sexual dysfunction. We thus aimed to assess, (i) whether there are significant positive associations between specific SE ratings and ratings on HDRS-17 items that overlap in content with these SEs (e.g., HDRS insomnia items and sleep-related SEs), (ii) whether such associations also impact the HDRS-17 sum-score, and (iii) whether outcome measures like the unidimensional HDRS-6 subscale and the HDRS-17 depressed mood item, which display little overlap with common antidepressant SEs, are less influenced by SE severity^3^.

## Methods

### Data acquisition

Adverse event and efficacy data from fifteen acute phase and placebo-controlled trials of duloxetine in major depression using HDRS-17^18,19^ were acquired through ClinicalStudyDataRequest.com. Ten of these were included in the FDA Approval Package for duloxetine and five were post-marketing trials. We confirmed that we had access to all relevant trials by searching the FDA Approval Package for duloxetine, the ClinicalStudyDataRequest website, Eli Lilly’s Clinical Study Results portal and Clinicaltrials.gov (for references see Supplementary material p 8). For one trial named in the FDA Approval Package (trial name: HMAG) data were not available. For two of the obtained trials (trial names: HMAH and HMAI) symptom-level HDRS-17 ratings were not available. All non-includable trials investigated sub-therapeutic doses (5–20 mg) of duloxetine. For seven studies, SSRI comparators were used as active controls. Whereas these treatment arms were included in all analyses, non-SSRI comparator arms were excluded.

### Identification and grouping of SEs

Adverse events were classified according to their lowest level Medical Dictionary for Regulatory Activities (MedDRA) term^20^ and initially grouped into five categories: sleep-, somatic anxiety-, sexual dysfunction-, gastrointestinal function-, and weight loss-related SEs by two authors (FH and SDØ). All adverse events present at endpoint in at least five patients were considered. Due to a low prevalence of weight loss-related adverse events (1.2%), this group was merged with the gastrointestinal function group. Adverse events not considered to belong to any of these five groups were excluded. For each SE group, a severity score was calculated by adding up the severities of all included SEs (mild SE: 1 point, moderate SE: 2 points, and severe SE: 3 points). Overall SE severity was computed by summing the severity scores for all four SE groups.

### Statistical analyses

Due to similar SE profiles (data not shown), duloxetine and SSRI-treated patients were analysed together^21^. Since SEs may lead to treatment discontinuation, and since subjects dropping out early have had less time to improve, including drop-outs in the analyses could result in associations between SE ratings and symptom ratings related to time rather than to content overlap between SE and HDRS-17 ratings. To avoid this confounder, the primary analyses were based on the observed cases (OC) population including only patients with an observation at study endpoint (usually week 8, see Supplementary table 1).

To confirm that the complaints reported as adverse events were at least partly actual SEs of the active treatment, we first assessed whether the included adverse events were more common among patients allocated to duloxetine or SSRI than to placebo by contrasting severity scores for all four SE groups individually, and for overall SE severity, using unequal variance t-tests. We additionally compared the active treatment and placebo groups with respect to the percentage of patients presenting with at least one relevant SE using the chi-square test of independence.

We then used analysis of covariance (ANCOVA) to assess whether specific SE groups associated with particular HDRS-17 items in a predictable manner (e.g., HDRS insomnia items and sleep-related SEs). For these analyses, only patients receiving active treatment (duloxetine or SSRI) were included. All models included study as a fixed effect and baseline severity on the corresponding dependent variable as a covariate. The dependent variables were (i) the sum of the three sleep items (HDRS-17 items 4–6: initial insomnia, middle insomnia, late insomnia), (ii) the somatic anxiety item (HDRS-17 item 11), (iii) the gastrointestinal symptoms item (HDRS-17 item 12), and (iv) the genital symptoms item (HDRS-17 item 14). For each model, the severity of the four SE groups (sleep-, somatic anxiety-, gastrointestinal function-, and sexual dysfunction-related SEs) were included as covariates.

We then assessed whether overall SE severity (i.e., the sum of the severities of the four SE groups) predicted endpoint scores on three HDRS-based outcome parameters: (i) HDRS-17-sum, (ii) the sum rating of the six items included in the unidimensional HDRS-6 subscale (HDRS-6-sum) (which includes the items depressed mood, guilt, work and interests, psychomotor retardation, psychic anxiety and general somatic symptoms, but not the symptoms corresponding to frequently occurring SEs), and (iii) the depressed mood item. This was done to explore, (i) whether any observed item-level associations significantly impact the sum of all HDRS-17 items, and (ii) whether such an influence may be reduced by using outcome measures showing less content overlap with SEs. As for the individual SE groups, this was done using ANCOVA models including actively treated patients only, but using the overall SE severity term rather than the four individual SE group severity terms. Prompted by a statistically significant association between overall SE severity and outcome when measured using HDRS-17-sum, but not HDRS-6-sum or depressed mood, we also assessed the relation between overall SE severity and outcome on the complementary set of HDRS items not included in the HDRS-6 (non-HDRS-6-sum). We also analysed HDRS-17-sum based remission rates (HDRS-17-sum endpoint score ≤7) using logistic regression. The logistic model used a binary distribution with a logit link and was otherwise identical to the ANCOVA models described above.

All analyses were repeated on the ITT population, with missing values imputed using last observation carried forward (LOCF) methodology; patients randomised to treatment but not having a post-baseline HDRS-17 evaluation were however not included. To assess whether the presence of SEs may be confounded with time under treatment when analysing the ITT population, we also ran an ANCOVA model on this population with time (week) of the last observation as the dependent variable. The model included protocol as a fixed factor, and baseline HDRS-17 and overall SE severity as covariates.

All analyses were carried out through remote desktop access to the Clinical Trial Data Transparency environment using SAS Software version 9.4 (SAS Institute, Cary, NC, USA). All statistical tests were two-tailed. Since most hypothesis tests are not independent—the OC population is a subgroup of the ITT population, HDRS-17 subscales and items are nested within the HDRS-17, and SE categories are nested within the overall SE category—we did not correct for multiple testing.

## Results

Details on the included studies are presented in the supplement (Supplementary table 1). In total 4768 patients participating in 13 trials were included. Of these, 3630 patients (76.1%), 1145 of whom were treated with placebo, had an HDRS-17 rating at study endpoint and thus constituted the OC population. 4647 (97.5%) patients, of which 1500 were treated with placebo, had at least one post-baseline HDRS-17 evaluation and were hence included in the ITT analyses.

The adverse events included in each of the four SE groups are shown in Table 1. Endpoint values for mean SE severity by SE group, mean overall SE severity and the proportion of participants reporting at least one SE at endpoint in patients given duloxetine/SSRI treatment or placebo, respectively, are shown in Table 2. Participants assigned to duloxetine or an SSRI displayed significantly higher severity for each individual SE group, as well as higher overall SE severity. Actively treated patients were also more likely than placebo-treated patients to display at least one SE from each SE group individually and at least one SE from any SE group.

**Table 1:** Adverse events included in each side effect group.

SE group	Included adverse events (n at endpoint)
Sleep-related	Insomnia (303), Middle insomnia (35), Initial insomnia (24), Insomnia exacerbated (17), Sleep disturbance (15), Sleep restless (9), Difficulty sleeping (8), Sleep decreased (8), Sleep disturbed (8), Sleeplessness (5)
Somatic anxiety-related	Dry mouth (499), Dizziness (152), Sweating (69), Lightheadedness (46), Sweating increased (44), Urinary frequency (44), Palpitations (40), Night sweats (29), Chest pain (19), Increased urinary frequency (18), Tachycardia (16), Tremor (shaking of hands) (22), Tremor of hands (21), Palpitation (12), Urinary urgency (11), Heart rate increased (10), Shakiness (10), Orthostatic hypotension (9), Dizzy (9), Perspiration excessive (8), Hot flushes (8), Urinary incontinence (7), Premature ventricular contractions (6), Shaking (6), Chest heaviness (5), Hyperhidrosis (5), Tingling (5)
Gastrointestinal function-related	Nausea (308), Constipation (268), Diarrhoea (152), Appetite decreased NOS (73), Decreased appetite (54), Gas (53), Indigestion (51), Heartburn (50), Weight loss (47), Vomiting (40), Flatulence (33), Loose stools (22), Appetite lost (21), Stomach cramps (20), Upset stomach (18), Dyspepsia (17), Bloating (15), Diarrhoea NOS (14), Anorexia (14), Stomach pain (13), Gastroesophageal reflux disease (13), Stomach ache (12), Abdominal pain (9), Obstipation (9), Abdominal cramp (8), Flatus increased (8), Stomach upset (8), Vomiting NOS (7), Abdominal cramps (6), Belching (6), Abdominal bloating (6), Gastroesophageal reflux (6), Increased bowel frequency (5), Loose bowels (5), Loss of weight (5), Emesis (5), Stomach flu (5)
Sexual dysfunction-related	Libido decreased (86), Anorgasmia (42), Ejaculation delayed (31), Loss of libido (25), Orgasm abnormal (18), Erectile dysfunction (15), Sexual dysfunction (14), Erectile disturbance (13), Erectile dysfunction NOS (12), Delayed orgasm (12), Inability to orgasm (9), Ejaculation failure (7), Impotence (7), Increased libido (5)

**Table 2:** Number of adverse events at endpoint stratified by treatment.

Side effect (SE) group	Placebo, *n* affected (%)	Placebo, mean severity (SEM)	Active treatment, *n* affected (%)	Active treatment, mean severity (SEM)	Placebo vs active treatment, OR (95% CI); *p*	*p*^A^
Sleep-related	74 (6.5%)	0.10 (0.012)	227 (9.1%)	0.15 (0.010)	1.46 (1.11 to 1.91); 0.007	0.003
Somatic anxiety-related	154 (13.5%)	0.20 (0.018)	531 (21.4%)	0.34 (0.016)	1.75 (1.44 to 2.13); <0.001	<0.001
Gastrointestinal function-related	193 (16.9%)	0.29 (0.022)	589 (23.7%)	0.40 (0.018)	1.53 (1.28 to 1.84); <0.001	<0.001
Sexual dysfunction-related	28 (2.5%)	0.04 (0.009)	175 (7.0%)	0.14 (0.012)	3.02 (2.02 to 4.53); <0.001	<0.001
≧1 of the above	360 (31.4%)	0.63 (0.036)	1070 (43.1%)	1.02 (0.033)	1.65 (1.42 to 1.91); <0.001	<0.001

SEM, standard error of the mean; ^A^*, p* for unequal variance *t* test.

**Fig. 1:**
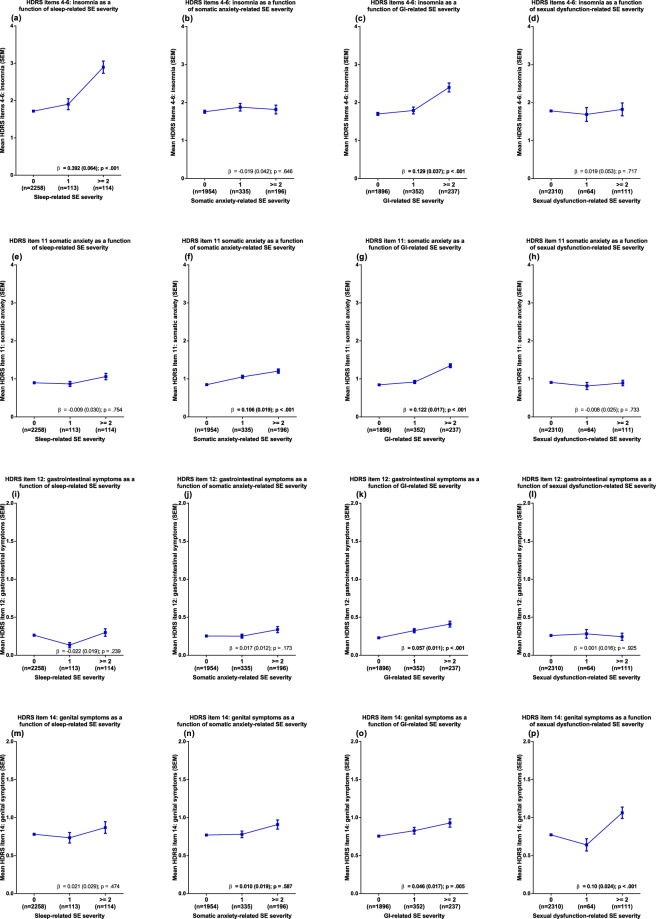
Endpoint HDRS-17 item ratings as a function of individual side effect (SE) group severity. Side effect severities present in ≤50 patients have been collapsed for easier visualisation. β Beta coefficient (SEM) for the specific SE-group predictor, GI gastrointestinal function, SEM standard error of the mean.

Associations between SE groups and HDRS-rated outcomes are displayed in Fig. 1a–p. While sleep-related SEs, somatic anxiety-related SEs, and sexual dysfunction-related SEs exclusively predicted the severity of corresponding HDRS-17 items (Fig. 1a, f, p), gastrointestinal function-related SE severity associated with all item-based outcome measures (Fig. 1c, g, k, o). The magnitude of the association with gastrointestinal function-related SE severity was on par with that of the somatic anxiety-related SE group for HDRS-17 item 12 (somatic anxiety) but three to four times less pronounced for HDRS items 4–6 (insomnia) and HDRS-17 item 14 (genital symptoms).

**Fig. 2:**
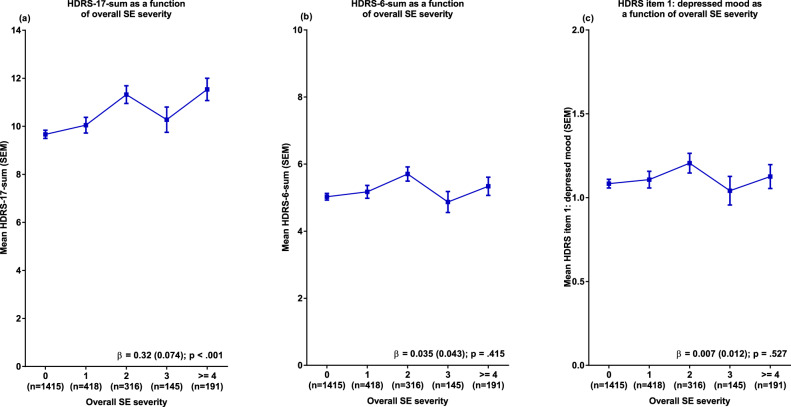
Endpoint HDRS-17-sum, HDRS-6-sum and depressed mood ratings as a function of overall side effect (SE) severity. Side effect severities present in ≤50 patients have been collapsed for easier visualisation. β Beta coefficient (SEM) for the overall SE severity predictor, SEM standard error of the mean.

**Fig. 3:**
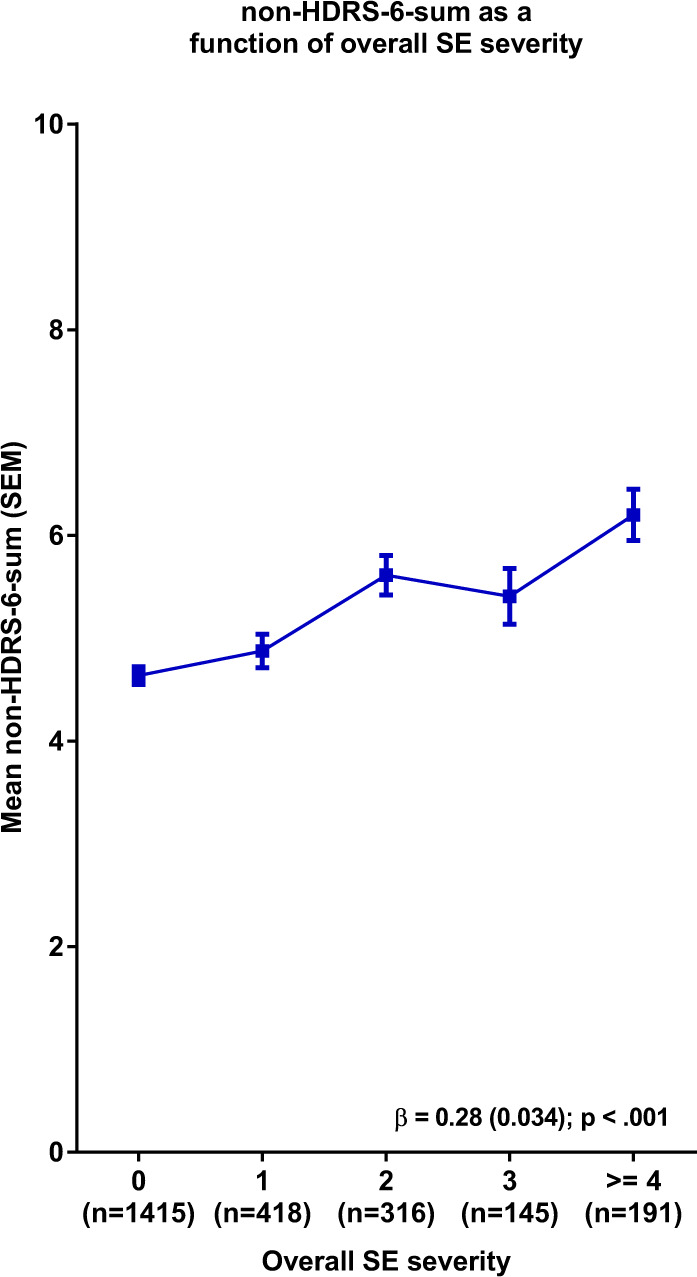
Endpoint non-HDRS-6 ratings as a function of overall side effect (SE) severity. Side effect severities present in ≤50 patients have been collapsed for easier visualisation. β Beta coefficient (SEM) for the overall SE severity predictor, SEM standard error of the mean.

While endpoint severity assessed using HDRS-17-sum was significantly impacted by overall SE severity, this was not the case when HDRS-6-sum or the depressed mood item was used (Fig. 2a–c). Follow-up analyses using the complementary set of HDRS-17 items not included in the HDRS-6 (non-HDRS-6-sum) as outcome parameter replicated the association observed between SEs and HDRS-17-sum ratings with a comparable strength of association (Fig. 3).

The positive association between overall SE severity and HDRS-17-sum was mirrored by a negative association between overall SE severity and HDRS-17-based remission rates (beta = −0.091 (0.027) and *p* < 0.001). While in total 41% of the actively treated sample achieved remission, this was the case for 44% of those with an SE severity score of zero, as compared to 41%, 32%, 37%, and 34%, of those with an overall SE severity score of one, two, three, or ≥ four, respectively.

### Sensitivity analyses

Sensitivity analyses of the ITT population were in line with those of the OC population (see Supplementary table 2 and Supplementary Figs. 1–3); however, significant associations between overall SE severity and HDRS-17-rated outcomes were more pronounced and numerous. Thus, in the ITT analyses, also HDRS-6-sum and depressed mood displayed significant associations with overall SE severity. Likewise, while in the ITT population all previously significant associations remained significant, the severity of somatic anxiety-related SEs predicted endpoint scores for HDRS item 12 (gastrointestinal symptoms) scores and the severity of sleep-related SEs predicted endpoint scores for HDRS item 14 (genital symptoms). The inverse association between overall SE severity and HDRS-17-based remission rates was observed also in the ITT population (beta = −0.137 (0.024) and *p* < 0.0001). While in total 35% of the active treatment sample achieved remission, this was the case for 39% of those with an overall SE severity score of zero, as compared to 36%, 28%, 31%, and 22%, of those with a score of one, two, three, or ≥ four, respectively. As hypothesised, overall SE severity was negatively associated with time under treatment in the ITT population (beta −0.16 (0.018), *p* < .001, and Supplementary figure 4).

## Discussion

The primary finding of this study is that the HDRS-17 sum-score associates with SEs commonly occurring in patients treated with SSRIs or SNRIs, and that this impact is primarily mediated by the HDRS items that show content overlap with the corresponding SEs. We thus report, (i) that the severity of a priori defined SEs, as expected, is higher at endpoint in patients receiving active treatment than in patients receiving placebo, (ii) that the severity of each individual SE group associates positively with corresponding HDRS item in patients given active treatment, and (iii) that there were no associations (in the OC population), or more modest associations (in the ITT population), between efficacy and SEs, when efficacy was measured using the HDRS-6 subscale or the depressed mood item.

As a follow-up analysis, we looked at the association between SEs and outcome measured by the sum of the HDRS items not included in the HDRS-6 subscale (Fig. 3 and Supplementary Fig. 3). These analyses largely replicated the associations seen with HDRS-17-sum. Together, these findings may partly explain the greater sensitivity to change that has been demonstrated for the HDRS-6 subscale and the depressed mood item when compared to HDRS-17-sum^17,22^, and further suggest that the antidepressant efficacy of SSRIs and SNRIs may have been consistently underestimated in previous meta-analyses using HDRS-17-sum as effect parameter^23–25^.

Since antidepressant SEs tend to be most impactful during the first days or weeks of treatment, we assumed that there may be confounding between SEs and time under treatment in the ITT population and hence considered the observed cases population as primary. This assumption was supported by the observation of an inverse relation between overall SE severity and time under treatment (Supplementary Fig. 4). On the other hand, due to differential dropout, it is likely that the observed cases population is depleted with respect to patients who have had the worst response to treatment (due to, e.g., lack of efficacy or intolerable SEs). Such a dropout mechanism should lead to an underestimation of the impact of SEs on HDRS-17-rated efficacy. In line with this, associations between overall SE severity and HDRS-17-rated outcomes were stronger in the ITT population, and there were also two additional non-specific associations between individual SE groups and HDRS item-based outcome parameters in this population (Supplementary Figs. 1–3).

When interpreting the present data, it should be considered that an association between SEs and corresponding HDRS-17 items may not only reflect actual SEs impacting the rating, but could also be caused by symptoms of depression being erroneously reported as SEs. As the included SEs are more common on active treatment, and since most of them can be elicited, at similar frequency, also in non-depressed subjects exposed to molecules inhibiting serotonin reuptake^26^, it however seems likely that the covariation to a considerable extent be indeed due to SEs influencing the HDRS-17. The possibility that certain symptoms, and/or patients with certain symptom constellations, are less likely to improve, or more likely to deteriorate, with antidepressant treatment, can however not be excluded.

In order to address a popular theory according to which the beneficial effect of antidepressants is due to SEs unblinding patients and raters, hence amplifying the placebo response in those on active treatment and/or bias the severity ratings^27^, we have previously assessed the effect of side effects not otherwise specified on the evaluation of efficacy^28,29^. These studies however suggested SEs not to be a prerequisite for a robust difference between active drug and placebo to be at hand. The present results expand upon those of these previous publications by showing that also a number of specific and common SNRI- and SSRI-related adverse events are not associated with improved response with respect to depressed mood or other essential symptoms of depression.

This report focuses on the 17-item version of the Hamilton Depression Rating Scale (and subscales thereof), but there are many other instruments available to assess the severity of depression^30^. The HDRS-17 has been questioned for its comparatively strong focus on somatic symptoms – which may render it especially susceptible to picking up SEs of SNRIs and SSRIs – as well as for not including other important aspects of the depressive diagnosis^7^. While short HDRS-17-based subscales, with less emphasis on somatic symptoms, have been demonstrated to yield larger drug-placebo differences than the full HDRS-17^17,22^, more comprehensive rating instruments that include symptoms not rated by the HDRS-17 (e.g., irritability) have also demonstrated increased sensitivity^31,32^. To what extent severity assessments based on other depression rating scales are impacted by the presence of SEs remains to be investigated.

One could argue that SEs impact the overall well-being of the patient and that a rating scale including them provides a more accurate reflection of the overall usefulness of a drug than a scale not capturing unwanted SEs^33^. While this reasoning applies for any scale aiming to measure overall well-being, HDRS and other depression rating scales are supposed to specifically measure depressive severity. Not least for future drug development, it is important to be able to distinguish drugs that are highly effective against depression, but provoke SEs in some patients, from those that are highly tolerable but only modestly effective^34^. In this context, it is unfortunate that pharmaceutical companies and regulatory authorities still favour rating instruments with demonstrable shortcomings in this respect^4^.

This study has a number of limitations. First, the symptoms that may be captured by HDRS-17 item 11 (somatic anxiety) are many and diverse. While we used an inclusive definition for the somatic anxiety-related SE group, being more (or even less) restrictive with regard to which SEs to include may strengthen or weaken the association between this SE group and the various outcome parameters. Second, there may exist SEs of active treatment of sufficient intensity to influence HDRS-17 ratings, but not of sufficient impact as to merit an adverse event report (and/or vice versa). This should likely work to attenuate the observed associations between reported SEs and HDRS-17 ratings. Relatedly, since the HDRS-17 separates between loss of appetite (HDRS-17 item 12) and weight loss (HDRS-17 item 16), we originally intended to include weight loss as a separate SE category, but opted not to do so after noting that SEs coded as ‘weight loss’ had low prevalence at endpoint (1.2%). Given the well-known short-term weight decreasing properties of drugs inhibiting serotonin reuptake^26^, the low prevalence of weight loss-related SEs may reflect underreporting. Third, the association between adverse events and treatment response may also have been attenuated by the fact that both response and side effects should be influenced in the same direction by factors for which we did not control, such as inter-individual differences in dose, compliance, and drug metabolism. Fourth, due to the exploratory nature of this study we opted for a simple analysis scheme in which ‘mild’, ‘moderate’ and ‘severe’ SEs, irrespective of type, were translated into 1, 2 and 3 severity points, respectively, and then added together and analysed as linear predictors. This transformation, however, is questionable since severity categories are unlikely to be linearly spaced and since SEs likely differ in their association with HDRS-17 ratings. Thus, while we do believe that the results are strong enough to suggest a positive relation between SEs and (some) HDRS-17-derived outcome parameters, we do not assume this association to be linear. Fifth, early treatment emergent SEs^35^, as well as early worsening in symptoms associated with depression (e.g., irritability, anxiety and mania)^36^, have been shown to predict lower remission rates. For feasibility reasons we, however, restricted our analyses to efficacy and SE reports collected at the endpoint visit (or last post-baseline visit in the ITT population); assessing to what extent SEs that appear early in treatment but remit prior to endpoint visit affect response trajectories over time was thus beyond the scope of this project. Sixth, the impact of SE “double counting” likely varies between studies due to differences in rater training, retention rates and adverse event recording practices. Seventh, this is a secondary analysis of data collected during the clinical development of duloxetine. While potentially a strength that these studies were not aimed at assessing the extent to which SEs and efficacy ratings may co-vary, hence limiting expectancy effects, the results should be replicated in studies directly aimed at assessing these issues, as well as in studies of other classes of antidepressants and outcome measures.

In summary, while depressive severity as measured using the HDRS-17 depressed mood item or the HDRS-6 subscale is not impacted by common antidepressant SEs, the ratings of a number of other items included in the HDRS-17 do co-vary with SE ratings. These associations are predictable in the sense that side effects belonging to a particular domain (e.g., sleep or sexual dysfunction) primarily associates with HDRS-17 item ratings of the same domain. SEs may hence impact the assessment of efficacy when HDRS-17-sum is used as effect parameter, which suggests that studies and meta-analyses using the HDRS-17 as effect parameter may have underestimated the efficacy of SNRIs and SSRIs when used for the treatment of depression. These findings underline that it is high time for the HDRS-17 to be superseded by alternative measures of depression severity in drug trials^4^.

## Supplementary information

Supplementary materials
