# Correction: Do side effects of antidepressants impact efficacy estimates based on the Hamilton Depression Rating Scale? A pooled patient-level analysis

**DOI:** 10.1038/s41398-021-01403-w

**Published:** 2021-05-07

**Authors:** Fredrik Hieronymus, Alexander Lisinski, Elias Eriksson, Søren Dinesen Østergaard

**Affiliations:** 1 Department of Pharmacology, Sahlgrenska Academy, University of Gothenburg, Gothenburg, Sweden; 2 Department of Clinical Medicine, Aarhus University, Aarhus, Denmark; 3 Department of Affective Disorders, Aarhus University Hospital, Aarhus, Denmark

**Keywords:** Depression, Neuroscience

Correction to: *Translational Psychiatry*

10.1038/s41398-021-01364-0 published online 27 April 2021

The original version of this article unfortunately contained a mistake. During the review process the title of the article was changed but the authors forgot to update the title given in the Supplementary Material. The original article has been corrected.

## Supplementary information

Supplementary Materials

