# Supplementary material for: Do side effects of antidepressants impact efficacy estimates based on the Hamilton Depression Rating Scale? A pooled patient-level analysis
Source: Transl Psychiatry. 2021 Apr 27;11:249. doi: 10.1038/s41398-021-01364-0 (PMC8079707; doi:10.1038/s41398-021-01364-0)
Supplement: Supplementary file 1 — Supplementary materials [file 41398_2021_1364_MOESM1_ESM.docx]

**<**

**Do side effects of antidepressants impact efficacy estimates based on the Hamilton Depression Rating Scale? A pooled patient-level analysis**

**Supplementary information**

**Hieronymus *et al*.**

**Supplementary table 1: Included trials**

**Supplementary table 2: Number and mean severity of adverse events at endpoint, stratified by treatment (intention to treat).**

**Supplementary figure 1: Endpoint HDRS item ratings as a function of individual side effect group severity (intention to treat)**

**Supplementary figure 2: Endpoint HDRS ratings as a function of overall side effect severity (intention to treat).**

**Supplementary figure 3: Endpoint non-HDRS-6 ratings as a function of overall SE-severity (intention to treat).**

**Supplementary figure 4: Week of last observation as a function of overall side effect severity (intention to treat)**

**References**

**Supplementary table 1: Included trials**

| **Trial** | **Years conducted** | **Patients randomized** | **Treatments** | **Acute-phase duration** | **Mean age (SD)** | **N (%) female** | **N (%) with visit at week 8** |
| --- | --- | --- | --- | --- | --- | --- | --- |
| HMAQa^1^ | 1999 – 2000 | 173 | Duloxetine; Fluoxetine; Placebo (2:1:2) | 8 weeks | 41.4 (11.7) | 111 (64) | 114 (66) |
| HMAQb^2^ | 1999 – 2001 | 194 | Duloxetine; Fluoxetine; Placebo (2:1:2) | 8 weeks | 40.4 (11.2) | 129 (66) | 128 (66) |
| HMATa^3^ | 2000 – 2001 | 354 | Duloxetine; Paroxetine; Placebo (2:1:1) | 8 weeks | 43.7 (14.6) | 217 (61) | 250 (71) |
| HMATb^4^ | 2000 – 2001 | 353 | Duloxetine; Paroxetine; Placebo (2:1:1) | 8 weeks | 40.5 (11.5) | 217 (61) | 222 (63) |
| HMAYa^5^ | 2000 – 2002 | 367 | Duloxetine; Paroxetine; Placebo (2:1:1) | 8 weeks | 43.4 (11.2) | 267 (73) | 321 (87) |
| HMAYb^6^ | 2000 – 2002 | 392 | Duloxetine; Paroxetine; Placebo (2:1:1) | 8 weeks | 45.2 (11.0) | 273 (70) | 357 (91) |
| HMBHa^7^ | 2000 – 2001 | 245 | Duloxetine; Placebo (1:1) | 9 weeks | 42.4 (13.1) | 163 (67) | 173 (71) |
| HMBHb^8^ | 2000 – 2001 | 267 | Duloxetine; Placebo (1:1) | 9 weeks | 40.9 (13.7) | 184 (69) | 171 (64) |
| HMBV^9^ | 2003 – 2004 | 311 | Duloxetine; Placebo (2:1) | 8 weeks | 72.8 (5.6) | 185 (60) | 252 (81) |
| HMCB^10^ | 2002 | 282 | Duloxetine; Placebo (1:1) | 7 weeks | 40.5 (13.5) | 184 (65) | 192 (68) |
| HMCR^11^ | 2003 – 2005 | 684 | Duloxetine; Escitalopram; Placebo (2:2:1) | 8 weeks | 42.3 (12.4) | 446 (65) | 510 (75) |
| HMFA^12^ | 2006 – 2009 | 370 | Duloxetine; Placebo (2:1) | 12 weeks | 72.9 (5.9) | 234 (63) | 294 (80) |
| HMFS^13^ | 2007 – 2008 | 776 | Duloxetine; Placebo (2:1) | 12 weeks | 43.6 (12.2) | 489 (63) | 646 (83) |

Two studies, HMAH^14^ and HMAI^15^, lacked item-level HDRS data and could not be included. SD = standard deviation. ^1-15^ references found at the end of the supplement.

**Supplementary table 2: Number and mean severity of adverse events at endpoint, stratified by treatment (intention to treat).**

| **Side effect (SE) group** | **Placebo, n affected (%)** | **Placebo,**  **mean severity**  **(SEM)** | **Active treatment,**  **n affected (%)** | **Active treatment, mean severity (SEM)** | **Placebo vs active treatment,**  **OR (95% CI); p** | **p^A^** |
| --- | --- | --- | --- | --- | --- | --- |
| Sleep-related | 110 (7.3%) | 0.12 (0.013) | 315 (10.0%) | 0.17 (0.010) | 1.41 (1.12 – 1.76); .003 | .003 |
| Somatic anxiety-related | 205 (13.7%) | 0.21 (0.016) | 718 (22.8%) | 0.38 (0.016) | 1.87 (1.58 – 2.21); < .001 | <.001 |
| Gastrointestinal function-related | 261 (17.4%) | 0.32 (0.022) | 827 (26.3%) | 0.51 (0.020) | 1.69 (1.45 – 1.98); < .001 | <.001 |
| Sexual dysfunction-related | 32 (2.1%) | 0.037 (0.007) | 223 (7.1%) | 0.15 (0.011) | 3.50 (2.40 – 5.09); <.001 | <.001 |
| >= 1 of the above | 481 (32.1%) | 0.69 (0.034) | 1443 (45.9%) | 1.21 (0.035) | 1.79 (1.58 – 2.04); <.001 | <.001 |

SEM = standard error of the mean, ^A^ = p for unequal variance t test.

**Supplementary figure 1: Endpoint HDRS item ratings as a function of individual side effect group severity (intention to treat)**


Side effect severities present in $\leq$ 50 patients in the primary population have been collapsed for easier visualization and comparison. β = beta coefficient (SEM) for the specific SE-group predictor. GI = gastrointestinal function, SEM = standard error of the mean.

**Supplementary figure 2: Endpoint HDRS ratings as a function of overall side effect severity (intention to treat).**

Side effect severities present in $\leq$ 50 patients in the primary population have been collapsed for easier visualization. β = Beta coefficient (SEM) for the overall SE severity predictor. SEM = standard error of the mean.

**Supplementary figure 3: Endpoint non-HDRS-6 rating as a function of overall side effect severity (intention to treat).**

Side effect severities present in $\leq$ 50 patients in the primary population have been collapsed for easier visualization. β = Beta coefficient (SEM) for the overall SE severity predictor. SEM = standard error of the mean.

**Supplementary figure 4: Week of last observation as a function of overall side effect severity (intention to treat)**

Side effect severities present in $\leq$ 50 patients in the primary population have been collapsed for easier visualization. β = Beta coefficient (SEM) for the overall SE severity predictor. SEM = standard error of the mean.

**References**

1. EliLilly. Clinical Study Summary: Study F1J-MC-HMAQa. Available from: <https://assets.contentful.com/hadumfdtzsru/3FeEMX77nykUwsguguMgae/4f385628fa4e88bf5ec62fca7aedc23d/Duloxetine-F1J-MC-HMAQ-_Study-Group-A_.pdf>. Accessed January 24, 2020.

2. EliLilly. Clinical Study Summary: Study F1J-MC-HMAQb. Available from: <https://assets.contentful.com/hadumfdtzsru/5FZ15aVRBKKsmKIoeSgsMO/b1b988aefbb263aabdb413af7f3534a7/Duloxetine-F1J-MC-HMAQ-_Study-Group-B_.pdf>. Accessed January 24, 2020.

3. EliLilly. Clinical Study Summary: Study F1J-MC-HMATa. Available from: <https://assets.contentful.com/hadumfdtzsru/1D76MTikKkeSIuOS4SgQcq/433330f08ad190a430f5d0f2140cb4b3/Duloxetine-F1J-MC-HMAT-_Study-Group-A_.pdf>. Accessed January 24, 2020.

4. EliLilly. Clinical Study Summary: Study F1J-MC-HMATb. Available from: <https://assets.contentful.com/hadumfdtzsru/5CF1Of1xBu0iwq8MWwEGOY/00f0112ecaebe9e28c5ace5c2c6087f4/Duloxetine-F1J-MC-HMAT-_Study-Group-B_.pdf>. Accessed January 24, 2020.

5. EliLilly. Clinical Study Summary: Study F1J-MC-HMAYa. Available from: <https://assets.ctfassets.net/hadumfdtzsru/1HL0AWoJtaa2WYimwwcEKq/2ea036815bca669bf62d40c469a3e870/Duloxetine-F1J-MC-HMAY-_Study-Group-A_.pdf>. Accessed January 24, 2020.

6. EliLilly. Clinical Study Summary: Study F1J-MC-HMAYb. Available from: <https://assets.ctfassets.net/hadumfdtzsru/yno94nVSNwwcgoeMmAwAS/14193d0e0ea8ae55239c618ca545d666/Duloxetine-F1J-MC-HMAY-_Study-Group-B_.pdf>. Accessed January 24, 2020.

7. EliLilly. Clinical Study Summary: Study F1J-MC-HMBHa. Available from: <https://assets.ctfassets.net/hadumfdtzsru/5631dUoo6AIEkk6aEoukGC/26bb0c33f9f23143a313c6a3a71e8c1e/Duloxetine-F1J-MC-HMBH-_Study-Group-A_.pdf>. Accessed January 24, 2020.

8. EliLilly. Clinical Study Summary: Study F1J-MC-HMBHb. Available from: <https://assets.ctfassets.net/hadumfdtzsru/269cS82aHOCQUI46IWWA82/0adc651617d670d85a97fbb0d0820465/Duloxetine-F1J-MC-HMBH-_Study-Group-B_.pdf>. Accessed January 24, 2020.

9. EliLilly. Clinical Study Summary: Study F1J-MC-HMBV. Available from: <https://assets.ctfassets.net/hadumfdtzsru/5dwajnpI5aGYGCSyWEaU82/fab4491efbd43e01b0a76e6621ca4852/Duloxetine-F1J-MC-HMBV.pdf>. Accessed January 24, 2020.

10. EliLilly. Clinical Study Summary: Study F1J-US-HMCB. Available from: <https://assets.ctfassets.net/hadumfdtzsru/13CZBb2pcGGwUWo8O2uCgW/31dfe1445c494c769d4d8bf0aa15de96/Duloxetine-F1J-US-HMCB.pdf>. Accessed January 24, 2020.

11. EliLilly. Clinical Study Summary: Study F1J-US-HMCR. Available from: <https://assets.ctfassets.net/hadumfdtzsru/4Y4CmsyeTYK8mcMSs8UO0K/53985d89c9366687f2db81ea51e5b57c/Duloxetine-F1J-US-HMCR.pdf>. Accessed January 24, 2020.

12. EliLilly. ClinicalTrials.gov result summary for F1J-US-HMFA. Available from: <https://clinicaltrials.gov/ct2/show/record/NCT00406848>. Accessed January 24, 2020.

13. EliLilly. ClinicalTrials.gov result summary for F1J-US-HMFS. Available from: <https://clinicaltrials.gov/ct2/show/NCT00536471>. Accessed January 24, 2020.

14. EliLilly. Clinical Study Summary: Study F1J-MC-HMAH. Available from: <https://assets.ctfassets.net/hadumfdtzsru/2Zhzid30KkuMsGGW8u6IUW/b32d812285654ae8f8ecda52292ea3b5/Duloxetine-F1J-MC-HMAH.pdf>. Accessed January 24, 2020.

15. EliLilly. Clinical Study Summary: Study F1J-MC-HMAI. Available from: <https://assets.ctfassets.net/hadumfdtzsru/3afvgeT2nKyccC2YyuOIks/a0900df35e75b2e8d26d6d34dedd46d2/Duloxetine-F1J-MC-HMAI.pdf>. Accessed January 24, 2020.
